# Discharge from the trauma centre: exposure to opioids, unmet information needs and lack of follow up—a qualitative study among physical trauma survivors

**DOI:** 10.1186/s13049-021-00938-7

**Published:** 2021-08-21

**Authors:** Jeanette Finstad, Olav Røise, Leiv Arne Rosseland, Thomas Clausen, Ingrid Amalia Havnes

**Affiliations:** 1 Division of Emergencies and Critical Care, Department of Research and Development, Oslo University Hospital, Postbox 4956, 0424 Nydalen, Oslo, Norway; 2 Institute of Clinical Medicine, University of Oslo, Oslo, Norway; 3 Norwegian Trauma Registry, Division of Orthopedic Surgery, Oslo University Hospital, Postbox 4956, 0424 Nydalen, Oslo, Norway; 4 Norwegian Centre for Addiction Research, University of Oslo, Postbox 1039, 0315 Blindern, Oslo, Norway; 5 National Advisory Unit On Substance Use Disorder Treatment, Division of Mental Health and Addiction, Oslo University Hospital, Postbox 4959, 0424 Nydalen, Oslo, Norway

**Keywords:** Traumatic injury, Post-discharge care, Outcomes, Pain management, Mental health, Opioids, Qualitative, Interviews

## Abstract

**Background:**

Physical trauma is associated with mortality, long-term pain and morbidity. Effective pain management is fundamental in trauma care and opioids are indispensable for treating acute pain; however, the use and misuse of prescribed opioids is an escalating problem. Despite this, few studies have been directed towards trauma patients in an early phase of rehabilitation with focusing on experiences and perspectives of health and recovery including pain and persistent use of prescribed opioids with abuse potential. To explore pre- and post-discharge trauma care experiences, including exposure to opioids, physical trauma survivors were recruited from a major trauma centre in Norway that provides the highest level of surgical trauma care.

**Method:**

Qualitative exploratory study. Individual semi-structured interviews were conducted among 13 trauma patients with orthopedic injuries, known to be associated with severe pain, six weeks post-discharge. The interviews were recorded, transcribed verbatim, and thematically analyzed with an interdisciplinary approach.

**Results:**

The overarching theme was that discharge from the trauma centre and the period that immediately followed were associated with feelings of insecurity. The three main themes that were identified as contributing to this was (a) unmet information needs about the injury, (b) exposure to opioids, and (c) lack of follow-up after discharge from the hospital. Participants experienced to be discharged with prescribed opioids, but without information about their addictive properties or tapering plans. This, and lack of attention to mental health and psychological impact of trauma, gave rise to unmet treatment needs of pain management and mental health problems during hospitalization and following discharge.

**Conclusion:**

The findings from this study suggest that in addition to delivery of high-quality biomedical trauma care, health professionals should direct more attention to psychosocial health and safe pain management, including post-discharge opioid tapering and individually tailored follow-up plans for physical trauma survivors.

## Introduction

Worldwide, physical trauma represents 10% of global mortality [1]. It is a major contributor to years lived in disability and it is recognized as a public health concern because of its significant impact on and cost to the individual and society [2, 3].

Traditionally, the evaluation of trauma care has primarily focused on mortality measurements [4]. However, as medical advancements have increased trauma survival rates, trauma care evaluations have gradually come to emphasize the significance of functional health, return to pre-injury lifestyle, and quality of life [5, 6]. The importance of self-reported health among patients has also been increasingly recognized [7]. Additionally, exploring patient experiences might help health professionals to evaluate interventions and/or therapeutic concepts [8].

Research has shown that, several years after the trauma, trauma survivors have reported more limited social and physical functioning, including physical pain, compared to the population norm [9]. In addition, trauma survivors are at increased risk of developing mental health problems, such as post-traumatic stress disorder (PTSD), depression, and anxiety [10–13].

Prognosis and long-term trauma outcomes are linked to a wide range of factors other than the severity of the injury, including socioeconomic status, age, and comorbidities [3, 14, 15]. Pain is an expected consequence of injury, at least in the short term, and orthopedic traumas are among the most painful [16]. In a prospective study of 233 patients with traumatic orthopedic injuries in the United States, 59% reported moderate to severe pain at the time of hospital discharge [17]. Effective pain management is fundamental to trauma care, and opioids are indispensable for treating acute pain. However, the use and misuse of prescribed opioids is an escalating problem, which has resulted in a national health crisis in the United States [18]. A study conducted in the United States indicates that more than 50% of trauma patients are discharged with an opioid prescription [19], reflecting both the severity of post-injury pain and the prescribing traditions. These patients are at risk of developing opioid use disorders, which are known to severely impact quality of life. Moreover, opioid treatment for chronic pain is both cautioned against and rarely effective [20]. Therefore, to improve total care and prognosis for trauma patients, it is essential to gain more knowledge about their experiences of opioid use.

The majority of the research about risk factors associated with trauma recovery patterns and functional outcomes has employed quantitative rating scales and questionnaires. Few qualitative studies have explored patient experiences of health and recovery following unintentional injury. The published studies have identified unmet injury-related information needs, suboptimal discharge planning, and insufficient follow-up as barriers for physical recovery [21–27]. Still, these findings, including experiences with prescribed opioids, needs to be explored further to provide nuanced knowledge about patient perspectives of trauma care. Therefore, this study aimed to explore patient experiences with and perspectives on pre- and post-discharge trauma care during the one to two months following discharge, including experiences related to opioid use.

## Methodology

This qualitative exploratory study is an independent sub-study of a large national study in Norway: “Injury Prevention and Outcomes following Trauma” (IPOT) [28].

### Context

Oslo University Hospital (OUH) is the major trauma centre in the South-Eastern Health Region in Norway, and where the participants of this study were recruited. It is comparable to what the American College of Surgeons Committee on Trauma describes as a Level 1 trauma centre, which is where the highest level of surgical care is provided. Some trauma patients are offered interdisciplinary therapy at rehabilitation clinics before they are discharged to home. The municipality-level general practitioners (GPs) are then responsible for further rehabilitation and follow-up [29].

### Participants and recruitment

The participants were all patients 18 years or older and able to consent, and who had experienced complex and/or severe surgical trauma. These traumas included one or more of the following three types of injuries: (1) more than one fracture of the extremities, spine, or pelvis, of which at least one of the fractures required surgical treatment, (2) isolated pelvic fracture with neurological injury (acetabulum or pelvic ring), and/or (3) isolated thoracic injury with more than two rib fractures. Opioids refers to all compounds that binds to opioid receptors and includes natural opioids (morphine and codeine), semi-synthetic opiates (e.g., oxycodone), and synthetic opioids (e.g., fentanyl, tramadol) [30]. Participants who had not received opioids in the 30 days prior to the accidental event were defined as opioid naïve. Opioid use was not an inclusion or exclusion criterion.

To ensure that the sample in this study reflected well-informed individuals with trauma care experience the participants were identified and recruited with purposive sampling [31]. Patients that met the inclusion criteria was first identified at the trauma centres daily trauma-meetings and then visited at the respective wards. After they received information about the study they agreed to be contacted after discharge to arrange for scheduled interviews.

Fourteen persons who met the inclusion criteria were enrolled in the study, but one was lost to follow-up. In addition, one re-hospitalized patient with pain experiences was included to pilot test the interview guide, and data from this patient was included in the analysis. An overview of 12 of the 13 participants’ injuries and surgical treatments was obtained with their consent from their medical journals at the trauma centre, but data was missing for one participant. The semi-structured interviews took place from March to June 2019 at the participants’ residence on an average of six weeks (range two-seven) after hospital discharge. Three of the interviews were conducted at local health institutions due to complicated injuries that required long-term residential care.

### Interview guide and data collection

An interview guide was developed in collaboration with two former trauma patients, pilot tested and adjusted accordingly. The interview guide consisted of five main themes and contained open-ended and probing questions about trauma care, experiences of pain, opioids, post-discharge follow-up, and quality of life after trauma. Each interview lasted 45–90 min and was audio recorded and transcribed verbatim. JF conducted the interviews. To ensure an open space within which participant perspectives could be prioritized and inter-subjective understandings achieved, the interview guide had a flexible design [32]. In the interest of enhancing validity, to access sensitive topics [33] and facilitate cross-case analysis some findings regarding motivation for and experiences of opioid use from the first interviews were engaged as vignettes in subsequent interviews with new participants [32].

### Analysis

The illness trajectory framework (Corbin and Strauss), which Halcomb and Davidson [34] have applied to traumatic injury, provides the theoretical framework for the study. Central to this framework is an emphasis on the bio-psychological impact of injury and an understanding that pre-injury factors affect recovery [35]. Within this framework, the trauma care continuum is understood as a trajectory that consists of multiple phases, beginning from the time before the injury and continuing through the acute and stable to recovery phases. The acute phase incorporates resuscitation and acute trauma care to treat injuries and prevent complications. Successful acute interventions will lead patients to enter a stable phase including varying degree of medical interventions to maintain the current health status [34]. According to Halcomb and Davidson [34], “health is likely to be sustained or enhanced for many survivors along the stable phase of the trajectory for many years” (p. 237). Still, at some point, recovery may deteriorate and patients may enter the unstable phase, which includes complications in all aspects of health [34]. In this study, participant experiences in the acute and stabilization phases were explored. The theoretical framework was engaged when planning the study and developing the interview guide. Questions regarding pre-injury factors and the different phases of the trauma trajectory were included in the interview guide as these factors may affect recovery. The framework influenced the analysis as we specifically focused on biological, psychological, and social aspects of the trauma care experiences.

The transcripts were processed and coded in NVIVO 11 [36]. A six-step inductive thematic approach guided the analysis [37]. JF conducted the initial coding, grouped the codes into broader preliminary themes of significance and reviewed and modified the themes in regular meetings with the co-authors. In the final refinement, JF and IH held regular discussions to reach consensus that resulted in three overarching themes with associated sub-themes were defined: (a) unmet information needs, (b) exposure to opioids, and (c) lack of follow-up after discharge. The emerging theme, “feeling unsafe/insecure”, evolved throughout the analysis. To ensure that different clinical perspectives were taken into account in the final steps of the analysis, all authors who have experience in anesthesiology, orthopedics, psychiatry, addiction, and primary health care participated in the discussions. The findings represent the end-point of the thematic analysis: to produce the report and give a full description of the themes, supported with empirical material in the form of quotations and in-depth descriptions.

## Results

All participants were involved in traumatic accidents and suffered severe injuries that required activation and reception of a trauma team, highly specialized treatment at a trauma centre, and access to trauma care. A description of the participants’ characteristics is presented in Table 1. Prior to the acute injury, 12 (92.3%) of the participants were opioid naïve. While hospitalized, all participants experienced severe pain, were treated with opioids and were also discharged from the hospital with a prescription for opioids. At the time of the interview, which occurred averagely six weeks after discharge from the trauma centre, eight participants (61.5) were still using opioids. The participants were admitted to the trauma centre for a mean time of 13.3 (range 2–43) days. Two participants were discharged directly home, and eleven were transferred to the local hospital or a rehabilitation centre before they returned home.

**Table. 1:** Participants demographics (n = 13)

Age, mean (range)	50 (19–78)
< 35, n (%)	4 (30.8)
35–50	2 (15.4)
50–65	4 (30.8)
> 65	3 (23)
Gender, male	11(84.6)
Living with partner	9 (69.2)
Living with children < 18 years	3 (23)
*Pre-injury work status*	
Full-time or studying	10 (76.8)
Working part-time	1 (7.8)
Retired	2 (15.4)
*Mechanism of Injury*	
Motor vehicle crash	5 (38.4)
Skiing	3 (23)
Other^a^	4 (30.8)
Missing	1 (7.8)
Injuries required surgery, n (%)	11 (84.6)
Days admitted to PACU^b,c^, mean (range)	2.5 (1–7)
Age, mean (range)	50 (19–78)

^a^Other includes: hit by falling object, fall and sledge ride accident

^b^*PACU* post-anesthesia care unit

^c^None of the participants required invasive ventilation support during their time at the PACU or were admitted to the Intensive Care Unit

All of the participants described major changes to their lives related to the unexpected physical trauma and its effects on their current life situation. When hospitalized many participants felt dependent on and safeguarded by hospital staff. This was described as a contrast to the time following discharge as they had to care for themselves.

The overarching theme was that the participants described the trauma course, from the time of injury to rehabilitation, as a complex experience in which they were exposed to different factors that contributed to feelings of what we, in this article, describe as “insecurity and unsafety”. This reflects our effort to translate from Norwegian to English a single term, *“utrygghet”.* In Norwegian, “utrygghet” encompasses both the physical risks associated with trauma, trauma recovery, and opioids, and the immaterial and subjective insecurities associated with an unpredictable and uncertain recovery process in which the participants’ perceived needs for information and follow-up care were not adequately met. This translation thus also reflects the three main themes that were identified as contributing to feelings of what the participants described as “utrygghet”: (a) unmet information needs, (b) exposure to opioids, and (c) lack of follow-up after discharge from the hospital.

### Unmet information needs

With respect to the acute phase to one to two months post-discharge, all of the participants recalled having experienced a lack of information from health professionals about the following three areas: (a) the injury and expected level of physical function, (b) psychological reactions to trauma and pre-existing premorbid mental disorders, and (c) opioid side effects and tapering.

The manner in which the health professionals communicated was also a central area of concern. For example, many participants did not understand the information that was shared with them when physicians used medical terminology and unfamiliar words. Henning described this as follows:

I had the impression that some of these attending physicians who are experts in their fields, they come in and explain your status and use foreign words that you have never heard before, which just leaves you with questions . . . Several times, I had to ask, “can you please explain it in a more lay person language”.

For Henning, an understanding of his injuries was essential. In particular, he wanted an explanation of his lung injury, because he had lung drainage and severe pain. Instead, he was met by physicians who used complicated language, which left him more confused and insecure. Also, Henning did not fully remember what information he had received at the trauma centre, which he believed was related to opioids and his experience of them profoundly compromising his memory.

Andrine described a different kind of communication barrier to having her information needs met. She felt that the physician who had informed her about her injuries crossed a line by trying to persuade her to quit her favorite hobby, which was also the source of her traumatic incident. She explained:

. . . he almost started to nag that I should quit or find a new sport . . . then I got a bit angry . . . and he (the doctor) tried to give information about the injured body part and stuff but it just did not work . . . I did not listen at all at that point. It is not his business what I do in my spare time.

The information about the physical trauma became unavailable to her after she blocked out what was said in response to what she perceived as her doctor’s inappropriate and insensitive approach. For her, her hobby was a significant source of recovery motivation, which made it even more difficult when he challenged and dismissed, rather than tried to understand, how important this hobby was for her.

André offers another example of physicians trying to provide information without knowing enough about the patient and, in this case, without knowing enough about the patient’s medical history. Most of the participants did not experience that current and pre-morbid mental health problems received significant attention at the trauma centre, but this had a particular meaning for André, who had struggled with severe depression. During trauma care, he was never asked questions about previous or current mental health status. Furthermore, he was uninformed that the injury could affect his mental health. André explained what he experienced at the time of discharge:

. . . the doctor told me . . . I don’t know if he meant it as a joke . . . “you are getting a lot of strong painkillers to take home and you can easily take an overdose and die” . . . he told me . . . “but you don’t have any plans of doing that”, he said . . . “no I don’t have any plans to do that”, I said . . . “But clearly, if you were someone who planned to do that, you have the opportunity with a large pack of pills in the house…”

André experienced that the physician was uninformed about his comorbid depression, which seemingly resulted in a misguided approach to communicating essential information. As a result, André experienced that he did not receive the information that he needed, which resulted in him not feeling comfortable opening up about his mental health history and concerns to the physician.

Overall, participants experienced that health care professionals provided little information about how mental health could be affected by traumatic injury. While admitted to the trauma centre, three participants were offered a psychiatric consultation, but André was not one of them.

The vast majority of the participants expressed that they first and foremost needed a detailed explanation of their injuries, treatment plans, and surgical interventions from their attending health professionals. They also desired information about the expected physical outcome of their injuries and the treatment and follow-up that they would receive following discharge from the trauma centre. All participants experienced that several physicians were involved in their treatment process. While some desired better continuity, others found this unproblematic, like Ben, who explained:

Everyone had different opinions on how things should be done . . . people handle tasks in different ways, so it did not bother me and if I asked I got an answer [. .] So, if you do not ask, then I do not think that you will get any further [answers] either.

Ben did not expect to be provided with information without asking; he took an active role, and he also described an experience of participation in the decision-making processes. Ulf, who suffered from severe lower extremity injuries and complicated fractures, did not experience involvement in the treatment process. During the acute phase, he was not provided with any information:

Nobody told me anything in the beginning, they did not inform me [. .] It happened twice that doctors tore off [the bandage] and said “tomorrow I will perform surgery”, but without it happening . . . and you get uncertain, when there is a professional fight going on in which the doctors seemingly disagree with each other.

Ulf experienced that his injury was more important to the doctors than he was, as he felt that the physicians were almost exclusively focused on the complicated fractures and challenging surgery. This made it difficult for him to participate in the treatment process.

A few participants experienced receiving incorrect information about their injuries. André provides an example:

I felt that there was poor information along the way . . . first, I heard that I had one fracture in my ankle, then I heard that there were two . . . and then one again . . . and the day that I got discharged from the hospital . . . then I was informed that I had had a fracture in my neck . . . and that was the first time that I had heard of that.

André felt that his physical health was in jeopardy and he worried that the severity of the neck fracture had worsened during the period that he was unaware of it. Repeated incidents of incorrect information also compelled André to question the competence of the health care system in general.

### Exposure to opioids

Most of the participants were opioid naïve before the injury, and their experiences of opioid effects were diverse and new. Opioids were experienced positively during hospitalization because of the considerable pain relief that they provided. Some participants described the opioid side effects as an experience of being heavily sedated by alcohol or illicit substances. Additionally, some described the opioids as generating feelings of well-being, calm, somnolence, and relaxation. Several participants expressed that the sense of well-being was so desirable that it was easy to understand why opioids are so addictive. When Henning was asked how he felt when he was given opioids, he responded:

Lethargic, relaxed, and intoxicated . . . I clearly felt like that when I was taking painkillers . . . Right away, I felt that these [opioids] were addictive . . . I felt that it was totally okay to be bedridden for a few weeks as long as I could feel intoxicated like that.

Henning sustained multiple rib fractures. Besides, he had an upper extremity fracture that caused extreme pain and immobilized him. To him, the feeling of being high on opioids made the severe trauma and its effects bearable. Early in the acute phase, he understood that he would be immobilized for weeks. Hence, he allowed himself to lie in bed and enjoy the feeling of being high. He compared the feeling to being in a bubble in which he felt absent and “comfortably numb”. On the other hand, Henning had mixed emotions about taking opioids. He knew that the analgesic effect was necessary and he enjoyed the pleasurable effect, but still, he wanted to stop using opioids because he understood that the enjoyable feeling could produce a desire to continue taking them.

The participants’ negative experiences with opioids during the hospitalization period were related to negative side effects. Almost all of the participants experienced side effects, including, most commonly, constipation, nausea, reduced respiratory rate, and dizziness. Fred described having experienced severe side effects, including visual and auditory hallucinations, paranoid symptoms, and psychedelic nightmares, which he interpreted as symptoms of having acquired a severe mental illness:

It was absolutely awful. I have never been so scared in my entire life . . . I did not dare to sleep because I felt that they were doing something to me . . . because they were attaching things to me and giving me electric shocks . . . It was terrible because I did not know . . . no one told me what they were doing to me . . . I was so incredibly scared.

Fred suffered paranoid symptoms and therefore neither trusted the hospital staff nor informed them about his experiences, resulting in what he described as a traumatic experience that continued after discharge. He had not been informed about this rare opioid side effect, and none of the health professionals at the trauma centre had asked him how he was feeling. He associated opioid use with paranoid symptoms, hallucinations, and anxiety. Although he suffered severe pain, he refused to take oxycodone and tramadol because he was terrified of returning to mental trauma. Subsequently, Fred struggled to understand why he had suffered hallucinations, paranoid symptoms, and severe nightmares.

A few participants also related feelings of shame to opioid use. This shame was triggered by memory loss and episodes during which the participants had experienced a lack of control. Fred, for example, vaguely recalled an episode in which he had misbehaved towards the nurses in the PACU. He remembered standing in bed, screaming, and then being yelled at and told that his behavior was unacceptable. He described feeling embarrassed and like he owed the nurses an excuse for his behavior. However, no one talked to him about the episode afterward.

#### Unmet needs for information about opioid use

Almost all of the participants experienced that nurses who administered their opioid treatment during hospital admission and physicians who prescribed opioids at discharge, did not provide information about side effects, addiction, or tapering. A few participants said that they did not expect information about opioids because they were already aware of the potentially harmful effects. Also, some said that information was easily accessible on the package leaflet. Others were surprised that health professionals administered strong, addictive medications without providing further information. Helge explained:

I can´t remember that they gave me information like “these are strongly addictive and you need to quit as soon as possible” . . . I have never been told that . . . I have been told that “if you have pain you should take pills because you´re not supposed to be in pain.”

Helge associated opioids with illicit substances and was aware of the risk of dependence. He preferred to withhold parts of his daily dose and feel pain rather than take what he feared could lead to dependence.

David provides another example of how physicians failed to provide information about opioids when he as a patient received a prescription at discharge, David explained how his experience affected him and his wife in the days that followed:

I was given 100 tablets [codeine 30mg + paracetamol 400mg], 4 per day . . . that was the message I got . . . nothing about the dangers or anything. I was a bit frightened by that . . . my wife was sitting here reading about everything and she was the one who mentioned it . . . in the drug leaflet it said that the recommended dose was maximum 3 grams per day . . . and I got 4 (laughs) . . . and she saw that my eyes were starting to turn yellow . . . then [ . . ] I did not want to do this anymore.

David and his wife experienced that they had to obtain information about opioids themselves. To them, David’s physical health appeared to have been jeopardized by the health professionals who had not provided them with appropriate information.

#### Tapering of opioids

At the time of discharge from either the trauma centre or local hospital, nine participants lacked a plan for tapering off opioids. The four participants who had a tapering plan had received it from physicians at the local hospital or rehabilitation clinic, implying that physicians at the trauma centre did not discharge any of the participants with a plan for or information about tapering off opioids. At the time of the interview, seven participants were taking opioids, but in lower doses than those that were prescribed upon discharge from the trauma centre.

Following discharge, the participants who lacked a tapering plan sought information from other sources, and several reported that the Internet was their primary source of information. They felt responsible for starting the process of tapering, but described this as a challenging process during which they experimented according to what they thought was best for them, but while feeling insecure. Fred provides an example:

It is something that I´m experimenting with . . . nobody has said anything to me . . . the only thing the doctor said was “don´t take more pills than it says on the medicine package” [. .] I´m just experimenting because I know I need to stop taking them . . . but I don’t think I will get addicted, I don’t think so . . .

Despite lacking a tapering plan, most participants had started to taper on their own. Different strategies were used; some stopped abruptly or tapered over the course of a few days while others tapered over an extended period. Some participants wanted to stop using opioids because they felt disappointed that they were still taking them. Some were also motivated to start tapering due to assumptions that opioids are physically harmful. These assumptions were informed by the Internet and television. Some participants tapered even though they were still in pain, as they felt better tolerating the pain than being on opioids. A range of non-pharmacological pain management strategies, solely initiated by the participants’ themselves, were also engaged, including most prominently relaxation and diversionary activities.

Several of the participants developed symptoms of tolerance. Even after short-term use, they experienced that they needed more opioids to achieve the same level of pain relief. They perceived this as concerning and as supporting their understandings of opioids as powerfully addictive agents. A few participants experienced severe withdrawal symptoms. Henning, for example, suffered from a chronic condition and had previously been prescribed large quantities and high doses of benzodiazepines. As a result, he had mental and bodily experiences of withdrawal, which he described as awful. Henning decided to withdraw completely four weeks after the traumatic injury, he explained:

When I was in that phase and decided that now I just have to quit everything [. .] then I started to taper and it was five days of pure hell. I understand why people become addicted and, like, start with heroin or other things, because I was lying in bed cold sweating in pain . . . I rarely cry, but, at that time, I was crying three nights in a row.

Henning knew that the period of withdrawal would be physically and mentally demanding, and that managing it together with his GP would help him to feel safeguarded. He described the support from his GP as crucial to successfully withdrawal. Still, he was the only participant who planned to taper in cooperation with his GP.

### Lack of follow-up after discharge from trauma centre

For most participants, hospital discharge marked the end of a period of constant care and the beginning of a new period in which they and, in some cases, their next of kin had to assume care responsibilities themselves. Most participants received minimal amount of information about the follow-up possibilities from the trauma centre, which led participants feel insecure about the injury recovery process several weeks after discharge. More than half of the study participants had not been in contact with their GP after discharge. For those who had, the GP was described as having been well informed about the injury, but themes such as mental health, pain experiences, opioid use, and the importance of tapering were not typically focal points. Moreover, most participants did not have access to specialists at the trauma centre during the one-to-two month post-discharge period, so their needs for information could not be directed there.

For some, the post-discharge period was more challenging than expected, as pain and limited physical functionality made activities of daily life problematic. Still, the main difference was the absence of health care and the lack of access to health professionals. Helge had suffered complicated injuries and required constant care during the first weeks of his admission at the trauma centre, which he described as having made him feel important and safeguarded. For him, returning home was challenging, and he expected but did not receive comprehensive follow-up:

But if a doctor who had access to my medical record had called and asked me some questions . . . if he had just read through the whole medical record and then naturally made some thoughts about what to ask me [. .] I think it is a little strange that no follow-up was planned . . . But I do not know, when I have been home for three days, why no doctor has called.

Prior to his injury, Helge had not been to his GP for twenty years. Following his injury, he did not understand why he would arrange to see his GP. Because no one had informed him of what follow-up he could expect after discharge from the local hospital, there was a considerable discrepancy between that which he expected and that which he received.

Henning lacked confidence about his ability to manage his injury during the recovery period, which he related to a lack of information about what exercises he could do and a fear of aggravating the injury by pushing himself too far. As he explained:

[Trauma patients] could get some simple guidelines that are kind of reassuring . . . a list of what´s perfectly normal with broken ribs . . . that you feel uncomfortable with sudden movements . . . as simple as that . . . like a guideline for dummies, so that you don’t have to Google stuff to be reassured in a way.

As described, Henning experienced an uncomfortable role shift. At the trauma centre, information was easily accessible, he could ask questions, and he felt safe and cared for. At home, he had to seek out information actively, and he did not know who to approach when he was insecure and needed informed reassurances regarding his recovery status, functionality, and treatment plan.

## Discussion

In this study, we included accidental trauma patients with injuries known to be associated with severe pain. We explored experiences with pre- and post-discharge trauma care. Results show that the participants experienced discharge from the trauma centre as a transition into feeling unsafe and insecure. Concerning pain management with opioids, the participants reported that they were discharged without tapering plans and with unmet information and treatment needs. This affected their physical, mental, and social wellbeing.

### Opioids and side effects

Almost all of the participants were opioid naïve before the traumatic injury. They were given opioids while hospitalized and they described diverse physical and mental side effects, particularly in the acute phase. At least one had experienced frightening hallucinations that were understood as opioid-induced. None of the participants reported having received information about side effects, and several were unaware that the symptoms that they experienced were known or typical side effects of opioids; thus, they did not inform the hospital staff. Nearly 80% of patients who use opioids experience at least one side effect [38, 39], particularly in the acute phase [40]. Approximately 25% of opioid naïve individuals experience cognitive impairment [41]. This and the emotional distress that is related to the injury may affect cognitive function [11]. Therefore, patients may not be able to sufficiently process and remember information given about opioid use and trauma, particularly in the acute phase.

### Lack of a tapering plan

All of the study participants were discharged with opioid prescriptions as part of their pain management treatment plans. The vast majority did not recall having received information about the addictive nature of opioids, the risk of withdrawal symptoms, or the need for a tapering plan. According to the literature, more than half of trauma patients receive an opioid prescription upon hospital discharge [19, 42]. This may already introduce a risk of dependence, because opioid tolerance can develop within only a few weeks of continued use [43]. Nevertheless, the study participants experienced that their trauma care was predominantly discontinued upon discharge. Those without tapering plans sought information on the Internet and experimented with dosage reduction due to a fear of developing dependence. Some of the participants also felt that their opioids might serve functions other than pain relief, such as a ‘time-out’ from a difficult life situation and temporary relief from symptoms of depression. Studies have shown that patients with chronic non-cancer pain and depression are more likely than those without depression to use opioids to alleviate symptoms that are not pain-related [44]. Additionally, Helmerhorst et al. [45] found that patients who continued using opioids one to two months after musculoskeletal trauma had higher pain scores, more psychological distress, and more disability than those who discontinued opioid treatment. Moreover, lack of pain management information and unclear discharge materials have previously been identified as a barrier to using analgesic regimens as prescribed [46]. Furthermore, chronic pain is correlated with depression [47].

### Mental health problems related to the injury

The participants commonly reported psychological reactions and emotional distress as a consequence of their traumas, including feeling depressed. This finding is consistent with previous studies showing that survivors of trauma have an increased risk of developing mental health problems [11, 21, 48, 49]. Studies have shown that trauma patients have increased risk for developing PTSD and associated comorbid conditions, such as anxiety and depression [10–13]. It has also been shown that 25% of trauma patients were diagnosed with a mood or anxiety disorder during the first three years post-injury [50]. At the same time, there is existing evidence that a mental health diagnosis may heighten the risk of opioid misuse [44]. Life-threatening experiences have been identified as a predictor of psychological reactions and PTSD [51], thus underscoring the need for health professionals to inform patients of this possibility and map psychological responses at an early phase. While admitted to the trauma centre only a minority of the participants received information about common psychological reactions to trauma and offered a psychiatric consultation. At discharge, physicians did not map pre-existing mental disorder or present mental health status, nor did they provide information about how to proceed and seek help if psychological challenges should follow. This finding is of significant concern, as studies have shown that, even years after the injury, trauma survivors have a heightened risk of hospital admission because of mental health concerns, as well as elevated suicide rates [52]. Furthermore, the literature clearly emphasizes the importance of offering mental health resources to survivors after physical trauma [11, 12]. All of the participants in this study experienced social, financial, and/or physical loss due to the trauma. According to Harms and Talbot [49], such losses are significant stressors, and they can exacerbate the mental health impact of the injury and/or create new psychological concerns. Overall, our findings emphasize the complexity of the trauma recovery process and raise concerns about the limited attention that the health professionals lent to post-trauma mental health and the minimal-to-no psychological support that the participants were offered.

### Communication and information

Poor communication on the part of the health professionals and the use of medical terminology were reported as barriers to receiving adequate information. High-quality communication may increase treatment adherence, improve recovery [53], and facilitate reciprocal interaction [54]. A few participants reported instances when physicians had provided information about their injuries, but failed to address their concerns about the physical outcomes. Braaf et al. [22] report similar findings and claim that unfavorable communication attributes can lead to distress in patients. To achieve successful reciprocal interaction, health professionals need to explore and understand the physical, mental, and social needs of their patients [55]. Besides, health professionals must address patient concerns and confirm that patients fully understand the information. Some trauma studies have identified individual health literacy, cognition, and self-efficacy as factors that affect how trauma patients access, use, and understand health information [22, 56]. Alberti and Nannini [57] claim that the most crucial way to achieve patient comprehension is to simplify the provided information. These factors and their impact on communication quality, as well as the need for information to be communicated in the first place, are essential for health professionals to consider.

### Unmet information needs

The vast majority of the participants had unmet needs for information about the severity and prognosis of the injury, trauma-related emotional distress, physical recovery expectations, and the follow-up care pathway. This finding is similar to those of other studies among severely injured patients [21, 24, 26]. Other studies have also found that inadequate information during hospitalization can lead to a higher incidence of pain and a lower health-related quality of life [58]. Besides, unmet information needs may lead to patient dissatisfaction and distress [22, 25], and can be a barrier that makes it difficult for patients to return to the pre-injury lifestyle, including physical, occupational, and social function [26]. Although participants viewed health professionals as responsible for providing information, some experienced that adequate information was not provided unless they assumed an active role and asked questions. According to Currie et al. [59], information quality can be affected negatively by “passive patients,” who deliberately avoid asking questions or do not report relevant information to health professionals. The participants in this study, however, lacked previous experience with severe injuries and did not know what to expect or what information they would desire. Kellezi et al. [24] describe similar findings from a study conducted among patients whose lack of knowledge about what to ask was identified as contributing to a lack of information. Upon discharge from the trauma centre, the participants desired tailored information with simplified explanations of the injury and treatment, including written guidelines and recovery-promoting exercises.

### Unprepared to return home

Participants commonly reported feeling unprepared for the challenges that they experienced following discharge to home. A lack of information about the follow-up care pathway and experiences of poor follow-up resulted in feelings of being unsupported and abandoned. This perception was most prominent among participants who went directly home, compared to participants who were first transferred to a rehabilitation centre, where the continued support of health professionals and immediate access to information might have made them feel more secure and, eventually, more prepared for the sudden contrast of returning home. In this study, some participants were health service naïve and lacked an understanding of how the health system worked. Besides, lack of information about the follow-up care pathway resulted in poor awareness of how to proceed and who to contact with respect to injury-related health concerns, including mental health problems. Other studies have highlighted the need for a better follow-up care pathway for trauma patients, as well as a follow-up contact to coordinate post-discharge care [25, 60]. Furthermore, structured discharge education has been shown to reduce pain, improve follow-up care compliance, and increase patient satisfaction six weeks after discharge [61]. Seamless transitions from trauma centres to primary care are an essential goal in trauma care. To achieve this, the revision of the “National traumaplan—trauma systems in Norway” includes recommendations for trauma patients to receive an individual plan of follow-up and further rehabilitation when discharged to primary care in the municipality [62]. Although our sample is small, our findings indicate that these recommendations are far from fulfilled in today’s practice.

### Strengths and limitations

This study generates new and detailed insight into patient experiences with pre- and post-discharge trauma care. A notable strength of this study is the heterogeneity of the participant sample, with respect to background, age, and injury type and severity. Limitations include the low number of participants and their recruitment from a single trauma centre. Because the inclusion criteria were narrow, the findings may not reflect the experiences of trauma patients with non-orthopedic injuries. The participants were interviewed a short time after discharge from the hospital which reduces the risk of recall bias. However, it is possible that the participants’ that had been at home for a longer period of time when the interviewed took place had more time to reflect on everyday life compared to those who were recently discharged. It is a possibility that accuracy and volume of particular memories were compromised by the effect of opioids and sedatives. Also, the hallucinations, paranoia, and derealization that the participants had interpreted as opioid-induced could have been caused by other factors, such as delirium, side effects, discontinued use of other medications or reaction to severe stress causing a chock-like state of mind.

### Clinical implications

Our findings point toward some critical implications for clinicians. First, there is a need to identify pre-injury and injury-related mental health problems to enable referral to specialized or interdisciplinary care. Second, health professionals need to provide information about the injury, common psychological reactions to accidental trauma and treatment options based on patient needs and comprehension. Additionally, the findings from this study highlight the need for nurses who administer opioids, and physicians who prescribe them, to provide information about common side effects, risk of dependence and the necessity of tapering. Verbal information may be reinforced with written guidelines and post-discharge access to a health professional at the trauma centre. Third, individually tailored plans for follow-up care in the municipalities should be integrated into trauma care. By establishing trauma outpatient clinics for routine consultation and follow-up, the ability to detect post-discharge challenges could be enhanced [11].

## Conclusion

This study shows that trauma patients may experience discharge from the trauma centre as a transition into insecurity. Unmet information needs, exposure to opioids and lack of follow up after discharge were identified as the three main themes contributing to this. Our findings also indicate that the primary focus of health professionals at the trauma centre seems to be the delivery of high-quality biomedical treatment for inpatients, and that more attention should be directed to psychosocial health and safe pain management, including post-discharge opioid tapering plans.

## Data Availability

Not applicable.
